# New Media Use and Mental Health of Married Women: Mediating Effects of Marital Quality

**DOI:** 10.3390/healthcare11212909

**Published:** 2023-11-06

**Authors:** Dong Zhou, Yi Xu, Qixuan He

**Affiliations:** School of Media and Communication, Shanghai Jiao Tong University, Shanghai 200240, China; dzhou002@sjtu.edu.cn (D.Z.); 0123hqxuan@sjtu.edu.cn (Q.H.)

**Keywords:** depression, new media use, marital satisfaction, married women, mental health

## Abstract

While previous studies have investigated the influence of new media on mental health, little is known about its effects on the mental health of married women. This is a crucial research area, given that married women commonly encounter distinct mental health difficulties. Also, current research fails to provide comprehensive, population-based studies, with most relying on cross-sectional designs. Therefore, this study aimed to investigate the relationship between new media use and mental health among married women in China, utilizing a nationally representative longitudinal dataset. We utilized a balanced panel dataset from 2016 to 2020 to establish a causal connection between internet use and the mental health of these women. Our findings indicate that internet use has a positive impact on the mental health of married women in China. Additionally, a structural estimation model (SEM) with 2020 wave data was utilized to investigate various new media use effects and explore mediating pathways of marital satisfaction. Consistently, there were negative findings between new media use, marital satisfaction, and depression. Furthermore, it was determined that new media usage had a significant negative impact on married women’s overall satisfaction with their spouses’ housework contribution, which, in turn, negatively affected marital satisfaction as a whole. The pathways that mediate the effect of marital satisfaction on depression differ across general internet use, streaming media use, and WeChat use. Examining various theoretical perspectives, we interpreted the indirect impact of new media use on mental health through marital satisfaction as passive mediation.

## 1. Introduction

Improving women’s well-being not only benefits household welfare, but also promotes sustainable social development. Existing literature has documented that women’s subjective well-being is declining and the new gender gap continues, with men having higher subjective well-being [1,2]. Statistically, the World Health Organization’s 2023 report suggests that an estimated 5% of adults worldwide suffer from depression, with women being more frequently affected than men [3]. When assessing the mental health of women, commonly considered factors include age, marital status, family relationships, education, career development, physical condition, and life events. However, the impact of new media usage, utilization of digital media and platforms distinct from traditional media, on the mental well-being of married women remains under-investigated.

By 2022, it is estimated that there will be 5.3 billion internet users worldwide, which represents 66% of the global population. As of 2022, social media has reached 3.96 billion users and continues to increase due to the growth of mobile device usage and mobile social networks in previously underserved markets [4]. Social media has become increasingly integrated into our experiences of family life. There have been qualitative changes in family relationships. However, the conclusions drawn from the research are inconsistent [5,6,7,8]. While there has been an increase in social media research, limited attention has been given to the impact on the health and family relationships of married women. This population is exceptionally susceptible to mental health challenges. This paper aims to investigate the relationship between the utilization of new media and mental health among married women, filling the knowledge gap, and evaluate mediating effects via the marital relationship.

Married women can utilize the internet for acquiring information and gaining social support, particularly during the pregnancy and postpartum periods [9,10]. Social media platforms offer the opportunity for married women to not only engage in personal leisure but also cultivate a sense of belonging while achieving professional success [11]. The study conducted by Schade et al. revealed that the use of information technology can heighten intimacy and overall satisfaction in relationships [12]. However, exposure to social media content has been linked to an increase in social comparison, which in turn has been associated with feelings of jealousy or lower perceived self-efficacy, ultimately leading to depressive symptoms [13,14,15]. Furthermore, McDaniel and Coyne have found that social media use can negatively impact intimacy and lower relationship satisfaction [16], while Coyne and colleagues report that video games can be a source of conflict and aggression among couples [17]. Previous investigations have often utilized cross-sectional designs or small sample sizes, which impede the capacity to establish causal relationships and generalize findings to broader populations [8]. To date, there is a significant dearth of longitudinal studies examining the influence of new media usage on married women. Our study advances knowledge by using nationally representative panel data for plausible causal inference and by addressing concerns about bidirectional correlation.

Note that the quality of marital relationships is a key factor to consider among married women. Our study is also innovative in exploring the intricate role of marital quality as a mediator in the relationship between new media use and the mental health of married women. Previous research has shown that the internet can disrupt marital boundaries and introduce distractions [18,19,20]. On the other hand, online communication, leisure, and entertainment activities have demonstrated potential in alleviating individual psychological stress, reducing conflicts within marriages, and enhancing marital satisfaction [21,22,23]. While existing research studies have provided valuable insights, they frequently draw inconsistent conclusions and have rarely explored various aspects of marital quality. This study examines the mediating role of marital quality and assesses two specific facets: satisfaction with spousal contributions to the economic and housework domains. Thus, this study contributes to a better understanding of the complex interplay among new media usage, marital quality, and mental health in this population.

Using a balanced panel dataset from 2016 to 2020, we identified an arguably causal relationship between internet use and depression among married women in China. Our results indicate that overall, internet use leads to lower levels of depression. Specifically, we found that internet users were less likely to experience depressive symptoms when compared to non-users. This finding can be interpreted by the uses and gratification theory [24]. Then, we adopted cross-sectional research designs and used the latest wave of 2020 to examine the impact of various new media platforms on changes in marital satisfaction and the mediating effects on depression levels. Strong evidence indicates that new media use corresponds with a direct and negative effect on depression levels, consistent with fixed-effect estimates. However, this effect does not alleviate depression through improvements in marital quality. We even discovered a passive mediating effect of new media use on mental health through lowering satisfaction towards spousal housework contribution, one of the main perspectives determining overall marital quality. Time displacement theory [16], changes in gender ideology [25], and changes in marriage expectations [26,27] are three potential explanations for this impactful mediator. All potentially lead married women to anticipate that their husbands will share more household chores. According to the marital discord theory [28], lower levels of spousal satisfaction indirectly cause concerns about depression.

## 2. Theoretical Background

### 2.1. New Media and Mental Well-Being

Over the past two decades, an increasing number of studies have investigated the relationship between new media use and subjective well-being, producing inconclusive findings [29,30,31]. Theoretically, the displacement hypothesis, which posits that time spent on one medium substitutes time spent on others, has been employed to explain the negative relationship between internet use and effects on subjective well-being [29]. Intensive internet use for communication displaces face-to-face interaction, leading to negative outcomes such as enhanced social isolation and decreased opportunities for social support and integration [32,33,34]. By contrast, the replacement hypothesis put forth by Gosling and Mason contends that internet usage has the potential to replace face-to-face social support and enhance overall well-being, especially for individuals with limited social abilities in face-to-face interactions or those residing in geographically isolated areas (such as immigrants or those who live far away from family and friends) [35]. This concept is partly supported by a collection of longitudinal studies that show loneliness leading to heightened internet usage, rather than the opposite [30,35]. 

Empirically, certain behaviors, such as cyberbullying and engaging in social comparisons, on social media are found to be associated with the development of depressive symptoms, particularly in young adults [36,37,38,39]. Paez et al. found insignificant relationships between internet use and subjective well-being after imposing multivariate controls in a longitudinal study [40]. Similarly, Jiang and Ngien found that Instagram use did not directly increase social anxiety [41]. The positive effects of internet use on mental health can be attributed to media coping, wherein online activities can alleviate stress-induced negative emotions by distracting from stressful encounters or allowing people to vent emotions [42,43,44]. Additionally, media literacy and education serve as valuable resources for individuals to enhance their well-being through better media navigation. By acquiring critical thinking skills and comprehending the possible impact of media on emotions and behaviors, individuals can make healthier and more knowledgeable decisions regarding their media consumption [45,46]. For example, the problematic effects of smartphone use can be alleviated via its educational purpose, ultimately promoting the well-being of young users [47].

In conclusion, it is essential to thoroughly consider the specific internet usage needs of individuals and provide more attention to married women. The majority of studies in this field have primarily focused on the general population or specific demographic groups, such as college students or adolescents [48,49]. However, there is inadequate research available concerning the female population. For instance, a recent study analyzed female residents in Egyptian villages and reported a significant negative correlation between addiction to Facebook and emotional regulation [50].

### 2.2. Marriage Quality and Mental Health of Women

The mental well-being of women is intricately tied to the benefits and challenges that arise from their familial and intimate roles. According to the marital discord model of depression, the risk of depression is significantly increased if the individual experiences marital discord or dissatisfaction [28]. This particular model asserts that marital discord or dissatisfaction can result in the decline of adaptive behaviors and an increase in negative behaviors, ultimately leading to depression. In distressed marriages, spouses often experience decreased levels of social support within their relationships, leading to a greater risk of depression. Coyne’s interpersonal theory of depression aligns with this perspective [51]. 

Distressed relationships often show increased levels of hostility, which predicts depression. Fincham et al. discovered gender-specific causal pathways in the correlation between depression and marital satisfaction through empiricism [52]. Causal paths from depression to marital satisfaction emerged among men, while among women it was in the opposite direction of marital satisfaction to depression. Recent research consistently shows that marital distress is a significant predictor of depressive symptoms [53,54,55,56,57,58]. Low marital satisfaction is a significant risk factor for depression, as observed in China [59]. Whisman et al. utilized the meta-framework of research triangulation by examining correlational, genetically informed, and intervention research on the link between relationship distress and depression [60]. Their extensive analysis has led to the conclusion that the current research body provides evidence supporting the assertion that relationship quality is a causal risk factor for depression. 

The prevailing literature consistently argues that lower marital satisfaction is a predictor of higher levels of depression in married women. Moreover, this study anticipated significant correlation between higher marital quality and improved mental health conditions. To our knowledge, prior research has focused mainly on overall marital quality and has not thoroughly investigated various dimensions of marital satisfaction from the perspective of both spouses. This study fills this gap by analyzing the mechanisms underlying marital satisfaction and distinguishing between satisfaction related to a spouse’s economic contribution and their assistance with housework.

### 2.3. New Media and Marital Satisfaction

The impact of information technology, especially social media, on marital relationships has gained significant attention. Previous studies concentrated on female participants in college environments [61] but there has been a shift towards investigating how interactive technologies affect the dynamics of relationships between married couples. 

This avenue of research has predominantly explored the challenges arising from technology use within marriages, encompassing issues like boundaries and distractions. For instance, studies have delved into topics such as how technology disrupts relationship boundaries [18,19,20] and causes distractions within the marital context [16,62,63,64,65]. Social media platforms like Facebook may decrease relationship satisfaction by displaying alternative romantic prospects and redirecting emotional investment from the committed relationship [66,67,68]. A study conducted on 143 women who were either married or cohabiting indicated that a majority of participants felt that technology devices, including computers, cellphones, smartphones, and TVs, frequently disrupted their interactions with their partners, such as during leisure time, conversations, and mealtimes. The study found that those who faced a greater number of technology interferences also reported experiencing higher levels of conflict over technology usage, lower relationship satisfaction, more depressive symptoms, and lower life satisfaction [16]. Yang and colleagues discovered that engaging in conversations with strangers online significantly decreased marital satisfaction in China [23]. Another study conducted in China revealed that broadband internet subscription had a strong positive impact on divorce rates in regions with low education levels, as well as in areas with higher income growth rates [69].

However, according to Yang et al., the use of the internet can improve marital satisfaction significantly. Against the backdrop of population mobility, the internet has been proven to increase the frequency of communication between geographically separated couples. Meanwhile, engaging in leisure and entertainment activities online can alleviate individual psychological stress, enrich cultural and spiritual lives, and promote a more positive emotional state. A similar study utilizing data from the Chinese Family Panel Studies (CFPS) indicated that utilization of the internet as a new media information access channel promotes marital satisfaction. The effect is more significant for young and middle-aged couples compared with elderly couples and more pronounced for urban couples compared with rural couples [21]. These internet-based activities predicted connection with family and friends, social support, and subsequently improved marital satisfaction, reduced marital conflicts, and better well-being for new mothers [22]. 

Interactive technologies have a dual effect for couples, as they promote connection while also causing distractions. These technologies provide the convenience for couples to communicate seamlessly even when apart. However, this very convenience may result in distractions that divert attention from crucial interactions between partners. A qualitative study examined the perception of couples towards interactive technology, such as cell phones, the internet, and social networking sites, in relation to their marital relationships. The study revealed the complex interplay between technology and relationships [70]. The study innovates by investigating the relationships between new media, spousal economic and housework contribution, marital satisfaction, and the mental health of married women in China.

## 3. Method

### 3.1. Study Design and Empirical Sample

Our study utilized data from the CFPS, a biennial panel survey of Chinese residents that is nationally representative [71]. The survey, which was launched in 2010, employed a multi-stage stratified sampling procedure with implicit stratification. Data from the latest wave, collected after July 2020, was used in our analysis. Our objective was to provide evidence for a plausible causal effect of internet use on the mental health of married women, using clear, concise language and a logical structure. A high-dimensional fixed-effect model was utilized to address endogenous issues arising from unobserved characteristics such as individual personality and bidirectional correlation. The analysis incorporated data from the most recent three waves, spanning from 2016 to 2020, and focused on commonly surveyed factors. A balanced panel dataset of 7068 married women was created using individual unique identification numbers. 

After establishing the fundamental relationship, we utilized structural equation modeling (SEM) to examine the mechanism of the impact of internet use on depression. In the 2020 wave of CFPS, richer information was surveyed. For instance, respondents were queried about their specific social media use and gave input on their satisfaction with their spouse. Marital quality survey questions were not posed in 2016. Thus, the study utilized data from the 2020 CFPS wave to investigate the mediating influence of marital satisfaction on the association between married females’ mental well-being and several forms of new media use. The sample for the study included 8073 married women.

### 3.2. Dependent Variable

Depression was measured using the Center for Epidemiologic Studies Depression Scale (CES-D) [72]. From 2016 to 2020, the comprehensive 20-item CES-D measure was administered. Participants were asked to rate how often they experienced symptoms associated with depression over the past week, such as restless sleep, loss of interest, and feeling lonely. Listed in this inventory are questions centered on emotional and mental state, including instances of unusual discomfort or lack of response to typical stimuli, poor appetite, inability to overcome negative feelings even with external support, difficulty focusing, depression, happiness, enjoyment of life, crying, sadness, and lack of motivation. Responses ranged from 1 to 4: 1 (not once or never), 2 (sometimes, 1–2 days), 3 (often, 3–4 days), and 4 (most of the time, 6–7 days). Responses ranged from 1 to 4, where 1 represented never, and 4 represented most of the time. Positive items were reversed, and the scores of all items were summed to measure the level of depression, with higher scores indicating higher levels of depression. The Cronbach’s α was approximately 0.95 for each year, and the Kaiser–Meyer–Olkin measure of sampling adequacy in the 2020 wave was 0.985.

### 3.3. Interested Explanatory Variables

*Internet use.* We used a dichotomous variable to measure whether respondents use the internet: 1 (*use the internet either* via *mobile or computer*) and 0 (*never use*). The 2020 wave of CFPS surveyed whether respondents used *WeChat* (a multi-purpose social media, messaging, and payment application) last year, and whether respondents used streaming media (including short video platforms such as *Kuaishou*, *Douyin*, *Weishi*, and *Douyin*, etc.) last week. Thus, we further created *WeChat use* and *Stream media use*, 1 (use) and 0 (never use).

### 3.4. Mediating Variables

*Marital satisfaction.* Three variables were used to assess marital satisfaction: Overall marital satisfaction, Satisfaction towards spousal housework contribution and towards spousal economic contribution. Respondents were asked “Are you satisfied with your overall marital relationship, with your spouse’s economic contribution to the family, with your spouse’s contribution in terms of sharing housework?” Respondents chose an answer from 1 (very unsatisfied) to 5 (very satisfied). Correspondingly, three ordinal variables are constructed. 

### 3.5. Covariates

Following the existing literature, we include a range of demographic characteristics: gender (1 = male, 0 = female), place of residence (1 = urban, 0 = rural), perceived social status (score 1–5 from the lowest to the highest), age (in years), years of schooling and self-rated health status (score 1–5, from the worst to the best). In all the estimations, we controlled for age, residential area, social status, education attainment, and health condition. 

## 4. Empirical Results and Discussion

### 4.1. Identify the Nature of Effect of Internet Use on Mental Health of Married Women

Table 1 reports the descriptive statistics of the total observations in the panel data. The average age of respondents was 48.5 years old. 74% lived in rural areas and 47.6% used the internet. Around 11% of this population had higher education certification. The average score of depression indicator is 33.72 for all married women. The fixed effect estimations are presented in Table 2. Each column represented one single regression and only estimates of interested were reported. 

Fixed effects regressions predicted within-person change before and after internet use, partialling out the individual-level unobserved contaminations. According to the estimates in Table 2, internet use was significantly and negatively associated with depression of married women, B = −0.513, *SE* = 0.172, *p* < 0.001, before controlling for covariates; B = −0.475, *SE* = 0.170, *p* < 0.001, after controlling for covariates. Internet use, in general, contributed to a higher level of welfare among married women. 

Although the existing literature documented complex results of internet use’s effects on well-being, our finding showed that the benefits of internet use, in general, outweigh its costs for Chinese married women. Social, leisure and entertainment activities conducted online can, to some extent, alleviate individual psychological stress, enrich people’s cultural and spiritual lives, and promote a more positive emotional state [23]. Chinese married women generally are occupied by family duties which make them live in a socially limited environment. The virtual community provides them space to communicate, entertain, win social support and release from role burden. The results can be supported by the uses and gratification theory [24] and positive suggestions of the placement theory. Internet can enable married women as individuals or members of their families as well as communities to increase control over the determinants of their mental health and thereby be in a position to improve their health status and health outcomes.

### 4.2. SEM Analyses of Mediating Effects of Marital Satisfaction

Table 3 reported the descriptive statistics of the sample of 2020 CFPS. There were 8073 married females at ages of 17 to 87. Around 72% of them were rural residents. Their average schooling year of education was 8, junior high education. The average score of depression index was 33.89, for all married women. Compared with satisfaction level of spousal economic contribution, evaluation of spousal housework contribution was significantly lower. Whereas, the level of overall marital satisfaction was higher than both observed marital perspectives, with an average score of 4.33.

**Figure 1:**
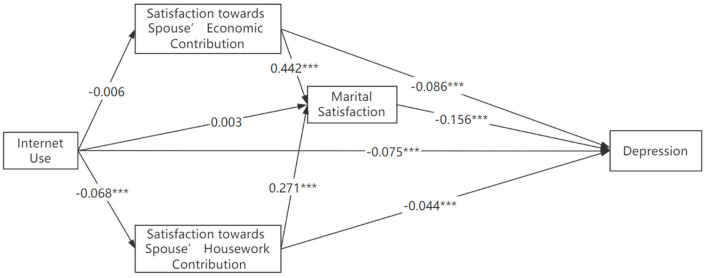
Mediating Effects of Factors related to Marital Quality. Note: *** *p* < 0.01.

Next, we analyzed the mediating effects of marital satisfaction with SEM (Figure 1) and maximum likelihood estimation method was used. The model fit indices show good fit: χ^2^ = 83.14, df = 6, CFI = 0.992, TLI = 0.958, RMSEA = 0.04, SRMR = 0.011. Internet use has a direct negative effect on depression, B = −0.075, *p* = 0.001, 95% CI = [−0.101, −0.049]. All three measures of marital satisfaction significantly and negatively predicted depression. Overall marital satisfaction significantly lowers the likelihood of being depressed, B = −0.156, *p* = 0.001, 95% CI = [−0.181, −0.131]. The two perspectives showed similar patterns with lower magnitude. These findings are consistent with the marital discord theory. Internet use was negatively associated with satisfaction towards spousal housework contribution, B = −0.068, *p* < 0.001, 95% CI = [−0.096, −0.041], while its effects on satisfaction towards economic contribution as well as the overall marital satisfaction were insignificant.

Meanwhile, both perspectives of satisfaction towards spouse significantly contributed to higher level of overall marital evaluation and further contributed to better mental health condition. Among the two perspectives, spousal economic contribution was relatively more important than the housework contribution in determining overall marital satisfaction (B = 0.44 vs. B = 0.27). Similarly, the spousal economic contribution was relatively more important than the housework contribution in lowering depression level (B = −0.086 vs. B = −0.044). 

The SEM results of our study also supported the validity of the marital discord model of depression in China, a large and collectivistic society. Consequently, these results, when considered in the context of the studies in Brazil [73] and Singapore [74], provide support for the “etic” perspective on marital satisfaction and depressive symptoms. This perspective posits that marital distress serves as a substantial predictor of depressive symptoms in both individualistic and collectivistic societies. In essence, marital distress consistently emerges as a risk factor for the development of depressive symptoms across different cultural contexts.

Model coefficients of interested reported in the path diagram are standardized. Robust model fit indices: *N* = 8073, χ^2^ = 83.06 df = 5, CFI = 0.992 TLI = 0.949, RMSEA = 0.04, SRMR = 0.011. Covariates among mediators are considered. * *p* < 0.05, ** *p* < 0.01, *** *p* < 0.001. MS1 = Satisfaction towards Spouse’ Economic Contribution, MS2 = Satisfaction towards Spouse’ Housework Contribution.

Model coefficients reported in the path diagram are standardized. Robust model fit indices: *N* = 8073, χ^2^ = 81.73 df = 5, CFI = 0.992 TLI = 0.950, RMSEA = 0.04, SRMR = 0.011. Covariates among mediators are considered. * *p* < 0.05, ** *p* < 0.01, *** *p* < 0.001. MS1 = Satisfaction towards Spouse’ Economic Contribution, MS2 = Satisfaction towards Spouse’ Housework Contribution.

Model coefficients reported in the path diagram are standardized. Robust model fit indices: *N* = 8073, χ^2^ = 82.2 df = 5, CFI = 0.992 TLI = 0.949, RMSEA = 0.044, SRMR = 0.011. Covariates among mediators are considered. * *p* < 0.05, ** *p* < 0.01, *** *p* < 0.001. MS1 = Satisfaction towards Spouse’ Economic Contribution, MS2 = Satisfaction towards Spouse’ Housework Contribution.

**Figure 2:**
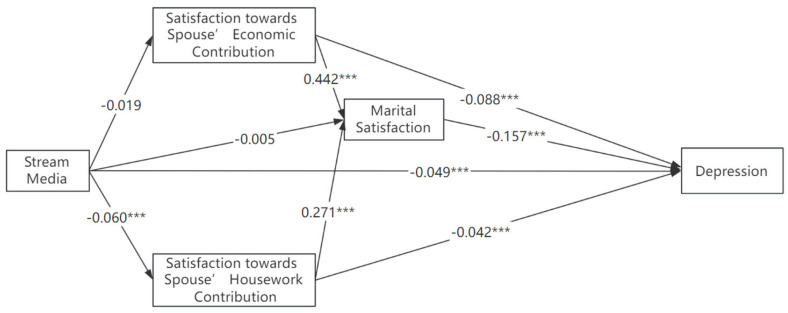
Alternative measure with Stream media use. Note: *** *p* < 0.01.

**Figure 3:**
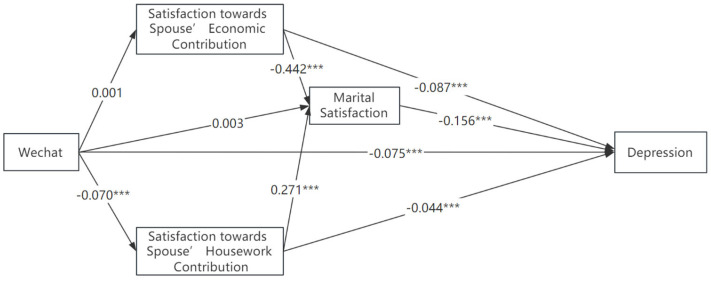
Alternative measure with WeChat use. Note: *** *p* < 0.01.

Alternatively, we used alternative new media platforms to make empirical analyses. The results are presented in Figure 2 and Figure 3. Similar conclusions are found when we use WeChat use or Stream media use as the key explanatory variable, respectively. The direct effect of streaming media use was significantly smaller than that of WeChat use (B = −0.049 vs. B = −0.075).

At last, we analyzed the indirect effects of new media with bootstrapping (replications 1000). Although Internet use has insignificant indirect effects on depression through marriage satisfaction, B = 0.005, *p* = 0.223, it has significant total effects on depression B = −0.071, *p* < 0.001. Internet use indirectly affects overall marital satisfaction through satisfaction toward spouse’s housework contribution, B = −0.016, *p* < 0.1. The insignificant indirect effects through marital satisfaction on depression could be attributed to the different effects caused by various internet use. Further analysis on the use of streaming media and WeChat yields a more detailed and nuanced understanding in this regard. 

Stream media use has a significant indirect effect on depression, B = 0.009, *p* < 0.01, and its total effect on depression is significantly negative, B = −0.040, *p* < 0.01. This suggests that using streaming media negatively affects the marital satisfaction, which leads to positive association with depression via the indirect route. Specifically, streaming media use is significantly associated with lower marital satisfaction regarding spousal housework contribution, which indirectly contributes to the lower overall marital satisfaction, B = −0.05, *p* < 0.01. This supports the time displacement hypothesis, suggesting that watching short videos may consume additional time that could have been allocated to housework. Consequently, this could result in conflicts related to the distribution of household tasks, whereas the total effect is mainly attributed to the negative direct effects on depression, B = −0.049, *p* < 0.001. Streaming media often offer a diverse range of entertainment content; such media enjoyment could alleviate the negative emotions [75].

WeChat use has an insignificant indirect effect on depression through marriage satisfaction, B = 0.005, *p* = 0.111, while its total effect on depression is significant, B = −0.070, *p* < 0.001. Similarly, WeChat use indirectly affects overall marital satisfaction via spousal housework contribution, B = −0.018, *p* < 0.05. The distinct patterns of indirect effects on depression associated with the use of streaming media and WeChat indicate the need to consider the specific ways in which individuals utilize the internet.

## 5. Discussion 

According to the existing literature, personality factors, health, religious commitment and spirituality, and socio-economic attributes such as income and financial and employment status are the most focusing determinants of subjective well-being [76]. Our results support that new information technology can serve as a new channel for helping married women enhance their mental health. Based on the fixed-effect estimates and the direct associations between internet use (streaming media use/WeChat use) and depression indicators, new media use demonstrates a significant negative effect on reducing depression. This is consistent with previous evidence suggesting that online communication, leisure, education, and entertainment activities have shown the potential to alleviate individual psychological stress [23,42]. Also, consistent with previous findings, marital satisfaction showed significant negative effects on depression. 

However, we did not find significant and strong evidence to support that this pathway can work through marriage. In fact, married women who use the internet exhibit significantly lower satisfaction towards spousal housework contribution. Satisfaction in spousal housework contribution, in general, constitutes a main component of the overall marital quality. Social media, to some extent, affects marriage quality through this channel indirectly and further influences the mental health of married females.

One potential reason is that the abundance of information/time on social media may also lead to time displacement, where the time spent on virtual interactions replaces time that could have been spent with one’s partner or on housework. This concept is rooted in the idea that time is limited and non-expandable, suggesting a trade-off between virtual and real-world relationships. McDanie & Coyne studied 143 married/cohabiting women and showed that the majority of participants perceived that technology devices (such as computers, cell or smartphones, or TV) frequently interrupted their interactions, such as couple leisure time, conversations, and mealtimes, with their partners [16]. The internet offers an array of ever-evolving computer-mediated communication platforms that allow individuals to connect with casual, professional, and romantic interests. It lowers their satisfaction towards spousal contribution to housework. In China, the top two social media platforms widely used are *WeChat* and *Douyin* (*TikTok*). The dominant function of *WeChat* is for communication and social interaction, while the latter platform is for entertainment and information. In 2022, the average daily usage time of users of *Douyin* is around 144 min, and the average number of user visits per day reaches about 20 times. Watching short videos displaces the time spent on relationship enhancing activities as well as time on housework. Resentment and dissatisfaction might be raised and lead to family conflicts. As presented in Figure 2 and Figure 3, the magnitudes of the direct effects of new media implicitly suggest that social interaction or communication are more likely to contribute to better welfare of married females.

A second potential reason is related to the changes in gender ideology. Nontraditional wives tended to report lower levels of marital quality [25,77,78]. Role conflict or role burden has been considered as a major source of stress for women expecting to combine work outside the home with that of family duties. Internet use can effectively mitigate discriminatory attitudes against females in the labor market and traditional gender norms in China [2]. While the female labor participation rate keeps increasing and the traditional division of labor within the family has gradually weakened due to information technology, married women are likely to expect their husbands to share more housework. We observed that social media use is not significantly related to the level of satisfaction with husbands’ economic contribution, but is significantly related to a lower level of satisfaction with husbands’ household contribution. Somehow, the findings accorded to social changes in China. 

In addition, social media use provides platforms for individuals to communicate with other users either privately or publicly. Fake identities or “the virtual perfect one” can cultivate perceptions or behaviors that facilitate behaviors that can be destructive to offline primary relationships [79]. Raised expectations toward a partner can lead to a higher level of dissatisfaction towards an offline spouse. Research has also shown a strong positive correlation between time spent on social media, particularly Facebook, and feelings of jealousy [80]. This relationship between jealousy and commitment aligns with the notion that more committed individuals are likely to experience stronger feelings of jealousy. It increases the dissatisfaction towards spouses. The intertwining of commitment and jealousy highlights the intricate dynamics between technology use and marital relationships.

These perspectives offer potential insights into the detrimental impact of social media on marital satisfaction. It is important to note that the passive mediating pathway leading to depression may vary in intensity depending on the specific social media platforms used. Therefore, further research is needed to explore the unique features and effects of different types of social media with more comprehensive measurements.

## 6. Limitations and Future Perspectives

We used the wide-ranging national representation afforded by CFPS to arrive at general conclusions in our study. However, we acknowledge certain restrictions arising from the survey design. Our measurements, especially those concerning internet usage, were relatively limited. For example, we utilized an indicator to establish whether respondents used the internet or not, as finer-grained data such as usage frequencies or specific functional purposes were lacking. To enhance understanding of the dynamics at play, a more comprehensive assessment of marital satisfaction that encompasses various factors is advisable. Additionally, incorporating nuanced measures on how new media use can influence relationships between married couples would offer insight into the underlying mechanisms. To bolster the robustness of our findings, future research should consider adopting these recommendations and including more comprehensive measurements.

While this study employed panel data to provide causal inference, the SEM analysis relied solely on 2020 data. Future research should incorporate longitudinal designs to capture dynamic changes and provide greater understanding of the evolving correlation between new media use and marital satisfaction. Additionally, experimental studies can be implemented to distinguish the combined effects of new media use. For example, further research could explore whether excessive time spent online reduces the opportunities for activities that enhance relationships, potentially leading to conflicts in familial roles.

Additionally, it is important to note that this study primarily drew data from China, and our findings can contribute to understanding the relationships in East Asian countries with similar social and cultural contexts. But the generalizability to western countries may be limited. Cultural, social, and economic factors may significantly impact the applicability of these research conclusions in different regions and countries. To increase the external validity of our research and provide a more thorough comprehension of the factors affecting women’s well-being, future studies should incorporate comparative analysis across multiple countries. Cultural context analysis can also be employed to elucidate the impact of cultural elements on the observed outcomes.

## 7. Conclusions and Implications

Our study delves into the intricate relationship between internet use, mental health, and marital quality among married women in China. Compared to the existing literature, we employed rigorous methodology by a longitudinal study through fixed-effects estimations for suggestive causal inference and using structural equation modeling to investigate the underlying mechanism. Our findings show that new media use in general supports married women’s mental health. Furthermore, new media use is negatively associated with satisfaction towards spousal housework contribution, but insignificant on satisfaction towards spousal economic contribution, two perspectives significantly associated with overall marital satisfaction. The findings bear significant implications for various aspects of individual and societal well-being.

Understanding the potential impact of new media use on marital satisfaction can inform interventions and strategies aimed at enhancing well-being in marital relationships. Therapists can include digital well-being components in counseling sessions to assist couples in navigating media consumption and improving their connection. Couples should be urged to openly communicate about their media use and its effects on their relationship. Balancing both online and offline activities is crucial to maintain healthy relationships. The study’s outcomes can assist individuals and professionals in the process of making well-informed decisions and implementing effective strategies to promote fulfilling and healthier marital relationships in the era of digital technology. Policymakers can refer to the research while formulating guidelines and regulations regarding media consumption.

## Figures and Tables

**Table 1:** Statistics Summary of 2016–2020 Married Women Sample.

Variable	Obs.	Mean	Std. Dev.	Min	Max
**Depression**	19,929	33.719	8.101	20	72
**Internet Use (1, Yes; 0, No)**	19,929	0.476	0.499	0	1
**Age**	19,929	48.543	13.409	16	87
**Residentials** (Rural = 1; Urban = 0)	19,929	0.744	0.437	0	1
**Health Condition (1–5)**					
1. Poor	19,929	0.191	0.393	0	1
2. Fair	19,929	0.157	0.363	0	1
3. Good	19,929	0.401	0.490	0	1
4. Very Good	19,929	0.139	0.346	0	1
5. Excellent	19,929	0.112	0.316	0	1
**Economic Social Status: Lowest–Highest 1–5 in local areas**					
1. the Lowest	19,929	0.090	0.286	0	1
2	19,929	0.157	0.364	0	1
3	19,929	0.458	0.498	0	1
4	19,929	0.172	0.377	0	1
5. the highest	19,929	0.123	0.328	0	1
**Schooling Years**	19,929	7.150	4.829	0	19

Note: all females married with spouse were followed from 2016–2020 CFPS.

**Table 2:** The Effect of Internet Use on Depression of Married Females Estimated from a Longitudinal Dataset.

Dependent Variable	(1)	(2)
	Depression	Depression
Internet	−0.513 ***	−0.475 ***
	(0.172)	(0.170)
Constant	33.303 ***	−4.967
	(0.091)	(9.886)
*Controls*	No	Yes
*Fixed Effects*	Yes	Yes
*N*	19,929	19,929
*R* ^2^	0.651	0.660

Note: Individual and time fixed effects are controlled, while covariates include age, age squared, residentials, education attainment, health condition and social status. Robust standard errors are clustered to individual level and reported in parentheses. *** *p* < 0.01.

**Table 3:** Statistics Summary of Married Women in 2020.

Variables	Obs.	Mean	SD	Min	Max
Age	8073	48.010	13.855	17	87
Rural Residents (Rural,1; Urban,0)	8073	0.720	0.449	0	1
Schooling Years	8073	7.676	4.932	0	22
Health Condition (1–5)					
1. Poor	8073	0.120	0.32	0	1
2. Fair	8073	0.145	0.35	0	1
3. Good	8073	0.440	0.50	0	1
4. Very Good	8073	0.119	0.32	0	1
5. Excellent	8073	0.176	0.38	0	1
Economic Social Status: Lowest–Highest 1–5 in local areas					
1. the Lowest	8073	0.073	0.260	0	1
2	8073	0.145	0.352	0	1
3	8073	0.465	0.499	0	1
4	8073	0.180	0.385	0	1
5. the highest	8073	0.136	0.343	0	1
Overall Marital Satisfaction (1–5, the least satisfaction to the highest satisfaction)					
1. very unsatisfied	8073	0.016	0.125	0	1
2. unsatisfied	8073	0.024	0.152	0	1
3. fair	8073	0.152	0.359	0	1
4. satisfied	8073	0.231	0.422	0	1
5. very satisfied	8073	0.577	0.494	0	1
Satisfaction towards Spouse’ Economic Contribution (1–5)					
1. very unsatisfied	8073	0.027	0.164	0	1
2. unsatisfied	8073	0.042	0.200	0	1
3. fair	8073	0.193	0.395	0	1
4. satisfied	8073	0.230	0.421	0	1
5. very satisfied	8073	0.507	0.500	0	1
Satisfaction towards Spouse’ Housework Contribution (1–5)					
1. very unsatisfied	8073	0.061	0.240	0	1
2. unsatisfied	8073	0.087	0.282	0	1
3. fair	8073	0.224	0.417	0	1
4. satisfied	8073	0.211	0.408	0	1
5. very satisfied	8073	0.416	0.493	0	1
Internet Use (Yes, 1; No, 0)	8073	0.598	0.490	0	1
Stream Media Use (Yes, 1; No, 0)	8073	0.480	0.500	0	1
WeChat Use (Yes, 1; No, 0)	8073	0.585	0.493	0	1
Depression	8073	33.889	8.191	22	72

## Data Availability

Publicly available datasets were analyzed in this study. This data can be found here: http://www.isss.pku.edu.cn/cfps/.

## References

[1] Stevenson B., Wolfers J. (2009). The Paradox of Declining Female Happiness. Am. Econ. J. Econ. Policy..

[2] Zhou D., Peng L., Dong Y. (2019). The Impact of Internet Usage on Gender Role Attitudes. Appl. Econ. Lett..

[3] World Health Organization, Mental Health Determinants and Populations, Department of Mental Health and Substance Dependence (2000). Women’s Mental Health: An Evidence Based Review.

[4] Dixon S.J. (2023). Social Media—Statistics & Facts. https://www.statista.com/topics/1164/social-networks/#topicOverview.

[5] Hertlein K.M. (2012). Digital Dwelling: Technology in Couple and Family Relationships. Fam. Relat..

[6] Stafford L., Hillyer J.D. (2012). Information and Communication Technologies in Personal Relationships. Rev. Commun..

[7] Carvalho J., Francisco R., Relvas A.P. (2015). Family Functioning and Information and Communication Technologies: How Do They Relate? A Literature Review. Comput. Hum. Behav..

[8] Dworkin J., Rudi J.H., Hessel H. (2018). The State of Family Research and Social Media. J. Fam. Theory Rev..

[9] Baker B., Yang I. (2018). Social Media as Social Support in Pregnancy and the Postpartum. Sex. Reprod. Healthc..

[10] Corno G., Villani D., de Montigny F., Pierce T., Bouchard S., Molgora S. (2023). The Role of Perceived Social Support on Pregnant Women’s Mental Health during the COVID-19 Pandemic. J. Reprod. Infant Psychol..

[11] Ho C.-H., Cho Y.-H. (2021). Social Media as a Pathway to Leisure: Digital Leisure Culture among New Mothers with Young Children in Taiwan. Leis. Sci..

[12] Schade L.C., Sandberg J., Bean R., Busby D., Coyne S. (2013). Using Technology to Connect in Romantic Relationships: Effects on Attachment, Relationship Satisfaction, and Stability in Emerging Adults. J. Couple Relatsh. Ther..

[13] Chrisler J.C., Fung K.T., Lopez A.M., Gorman J.A. (2013). Suffering by Comparison: Twitter Users’ Reactions to the Victoria’s Secret Fashion Show. Body Image.

[14] Chae J. (2014). Am I a Better Mother Than You?. Commun. Res..

[15] Ouvrein G. (2022). Mommy Influencers: Helpful or Harmful? The Relationship between Exposure to Mommy Influencers and Perceived Parental Self-Efficacy among Mothers and Primigravida. New Media Soc..

[16] McDaniel B.T., Coyne S.M. (2016). “Technoference”: The Interference of Technology in Couple Relationships and Implications for Women’s Personal and Relational Well-Being. Psychol. Pop. Media Cult..

[17] Coyne S.M., Busby D., Bushman B.J., Gentile D.A., Ridge R., Stockdale L. (2012). Gaming in the Game of Love: Effects of Video Games on Conflict in Couples. J. Abnorm. Psychol..

[18] Cravens J.D., Leckie K.R., Whiting J.B. (2012). Facebook Infidelity: When Poking Becomes Problematic. Contemp. Fam. Ther..

[19] McDaniel B.T., Drouin M., Cravens J.D. (2017). Do You Have Anything to Hide? Infidelity-Related Behaviors on Social Media Sites and Marital Satisfaction. Comput. Hum. Behav..

[20] Norton A.M., Baptist J., Hogan B. (2018). Computer-Mediated Communication in Intimate Relationships: Associations of Boundary Crossing, Intrusion, Relationship Satisfaction, and Partner Responsiveness. J. Marital Fam. Ther..

[21] Fan W.S., Wang S. (2022). Media as a “Marriage Mediator”: The Relationship between Media Information Access and Marital Satisfaction Based on Cfps Panel Data 2018–2020. Fujian Trib. (Humanit. Soc. Sci. Mon.).

[22] McDaniel B.T., Coyne S.M., Holmes E.K. (2012). New Mothers and Media Use: Associations between Blogging, Social Networking, and Maternal Well-Being. Matern. Child Health J..

[23] Yang H., Wang J., Xiao W., Liu Y. (2022). The Study in the Impact of Internet Use on Marital Satisfaction. Popul. J..

[24] Rubin A.M. (2002). Media Effects: Advances in Theory and Research.

[25] Davis S.N., Greenstein T.N. (2009). Gender Ideology: Components, Predictors, and Consequences. Annu. Rev. Sociol..

[26] Stevenson B., Wolfers J. (2007). Marriage and Divorce: Changes and Their Driving Forces. J. Econ. Perspect..

[27] Ye W., Sarrica M., Fortunati L. (2013). A Study on Chinese Bulletin Board System Forums: How Internet Users Contribute to Set up the Contemporary Notions of Family and Marriage. Inf. Commun. Soc..

[28] Beach S.R., Sandeen E., O’Leary K.D. (1990). Depression in Marriage: A Model for Etiology and Treatment.

[29] Huang C. (2010). Internet Use and Psychological Well-Being: A Meta-Analysis. Cyberpsychol. Behav. Soc. Netw..

[30] Song H., Zmyslinski-Seelig A., Kim J., Drent A., Victor A., Omori K., Allen M. (2014). Does Facebook Make You Lonely?: A Meta Analysis. Comput. Hum. Behav..

[31] Çikrıkci Ö. (2016). The Effect of Internet Use on Well-Being: Meta-Analysis. Comput. Hum. Behav..

[32] Kraut R., Patterson M., Lundmark V., Kiesler S., Mukophadhyay T., Scherlis W. (1998). Internet Paradox: A Social Technology That Reduces Social Involvement and Psychological Well-Being?. Am. Psychol..

[33] Pantic I. (2014). Online Social Networking and Mental Health. Cyberpsychol. Behav. Soc. Netw..

[34] Reinecke L., Aufenanger S., Beutel M.E., Dreier M., Quiring O., Stark B., Wölfling K., Müller K.W. (2016). Digital Stress over the Life Span: The Effects of Communication Load and Internet Multitasking on Perceived Stress and Psychological Health Impairments in a German Probability Sample. Media Psychol..

[35] Gosling S.D., Mason W. (2015). Internet Research in Psychology. Annu. Rev. Psychol..

[36] Bottino S.M., Bottino C.M., Regina C.G., Correia A.V., Ribeiro W.S. (2015). Cyberbullying and Adolescent Mental Health: Systematic Review. Cad. Saude Publica.

[37] Braghieri L., Levy R.E., Makarin A. (2022). Social Media and Mental Health. Am. Econ. Rev..

[38] Sherlock M., Wagstaff D.L. (2019). Exploring the Relationship between Frequency of Instagram Use, Exposure to Idealized Images, and Psychological Well-Being in Women. Psychol. Pop. Media Cult..

[39] Warrender D., Milne R. (2020). Social Media, Social Comparison and Mental Health. Nurs. Times.

[40] Paez D., Delfino G., Vargas-Salfate S., Liu J.H., Gil De Zúñiga H., Khan S., Garaigordobil M. (2019). A Longitudinal Study of the Effects of Internet Use on Subjective Well-Being. Media Psychol..

[41] Jiang S., Ngien A. (2020). The Effects of Instagram Use, Social Comparison, and Self-Esteem on Social Anxiety: A Survey Study in Singapore. Soc. Media Soc..

[42] de Wit J., van der Kraan A., Theeuwes J. (2020). Live Streams on Twitch Help Viewers Cope with Difficult Periods in Life. Front. Psychol..

[43] Neubaum G., Rösner L., Rosenthal-von der Pütten A.M., Krämer N.C. (2014). Psychosocial Functions of Social Media Usage in a Disaster Situation: A Multi-Methodological Approach. Comput. Hum. Behav..

[44] Wolfers L.N., Utz S. (2022). Social Media Use, Stress, and Coping. Curr. Opin. Psychol..

[45] Bullen M. (2007). Participation and Critical Thinking in Online University Distance Education. Int. J. E-Learn. Distance Educ..

[46] Supriyatno T., Susilawati S., Ahdi H. (2020). E-Learning Development in Improving Students’ Critical Thinking Ability. Cypriot J. Educ. Sci..

[47] Gui M., Gerosa T., Argentin G., Losi L. (2023). Mobile Media Education as a Tool to Reduce Problematic Smartphone Use: Results of a Randomised Impact Evaluation. Comput. Educ..

[48] Berryman C., Ferguson C.J., Negy C. (2018). Social Media Use and Mental Health among Young Adults. Psychiatr. Q.

[49] Sommantico M., Ramaglia F., Lacatena M. (2023). Relationships between Depression, Fear of Missing out and Social Media Addiction: The Mediating Role of Self-Esteem. Healthcare.

[50] Alenezi A., Hamed W., Elhehe I., El-Etreby R. (2023). Association between Facebook Addiction, Depression, and Emotional Regulation among Women. Healthcare.

[51] Coyne J.C. (1976). Depression and the Response of Others. J. Abnorm. Psychol..

[52] Fincham F.D., Beach S.R., Harold G.T., Osborne L.N. (1997). Marital Satisfaction and Depression: Different Causal Relationships for Men and Women?. Psychol. Sci..

[53] Beach S.R., Katz J., Kim S., Brody G.H. (2003). Prospective Effects of Marital Satisfaction Depressive Symptoms in Established Marriages: A Dyadic Model. J. Soc. Pers. Relatsh..

[54] Whisman M.A. (2007). Marital Distress and Dsm-Iv Psychiatric Disorders in a Population-Based National Survey. J. Abnorm. Psychol..

[55] Whisman M.A., Baucom D.H. (2012). Intimate Relationships and Psychopathology. Clin. Child Fam. Psychol. Rev..

[56] Whitton S.W., Stanley S.M., Markman H.J., Baucom B.R. (2008). Women’s Weekly Relationship Functioning and Depressive Symptoms. Pers. Relatsh..

[57] Overbeek G., Vollebergh W., de Graaf R., Scholte R., de Kemp R., Engels R. (2006). Longitudinal Associations of Marital Quality and Marital Dissolution with the Incidence of DSM-III-R Disorders. J. Fam. Psychol..

[58] Whisman M.A., Uebelacker L.A. (2009). Prospective Associations between Marital Discord and Depressive Symptoms in Middle-Aged and Older Adults. Psychol. Aging.

[59] Miller R.B., Mason T.M., Canlas J.M., Wang D., Nelson D.A., Hart C.H. (2013). Marital Satisfaction and Depressive Symptoms in China. J. Fam. Psychol..

[60] Whisman M.A., Sbarra D.A., Beach S.R.H. (2021). Intimate Relationships and Depression: Searching for Causation in the Sea of Association. Annu. Rev. Clin. Psychol..

[61] Baym N.K., Zhang Y.B., Kunkel A., Ledbetter A., Lin M.-C. (2016). Relational Quality and Media Use in Interpersonal Relationships. New Media Soc..

[62] Halpern D., Katz J.E. (2017). Texting’s Consequences for Romantic Relationships: A Cross-Lagged Analysis Highlights Its Risks. Comput. Hum. Behav..

[63] Morgan A.A., Landers A.L., Simpson J.E., Russon J.M., Case Pease J., Dolbin-MacNab M.L., Bland K.N., Jackson J.B. (2021). The Transition to Teletherapy in Marriage and Family Therapy Training Settings During COVID-19: What Do the Data Tell Us?. J. Marital Fam. Ther..

[64] Roberts J.A., David M.E. (2016). My Life Has Become a Major Distraction from My Cell Phone: Partner Phubbing and Relationship Satisfaction among Romantic Partners. Comput. Hum. Behav..

[65] Spencer T.A., Lambertsen A., Hubler D.S., Burr B.K. (2017). Assessing the Mediating Effect of Relationship Dynamics between Perceptions of Problematic Media Use and Relationship Satisfaction. Contemp. Fam. Ther..

[66] Clayton R.B., Nagurney A., Smith J.R. (2013). Cheating, Breakup, and Divorce: Is Facebook Use to Blame?. Cyberpsychol. Behav. Soc. Netw..

[67] Cravens J.D., Whiting J.B. (2015). Fooling around on Facebook: The Perceptions of Infidelity Behavior on Social Networking Sites. J. Couple Relatsh. Ther..

[68] Rahaman H.M. (2015). Romantic Relationship Length and Its Perceived Quality: Mediating Role of Facebook-Related Conflict. Eur. J. Psychol..

[69] Zheng S., Duan Y., Ward M.R. (2019). The Effect of Broadband Internet on Divorce in China. Technol. Forecast. Soc. Change.

[70] Vaterlaus J.M., Tulane S. (2019). The Perceived Influence of Interactive Technology on Marital Relationships. Contemp. Fam. Ther..

[71] Xie Y., Hu J. (2014). An Introduction to the China Family Panel Studies (Cfps). Chin. Sociol. Rev..

[72] Radloff L.S. (1977). The Ces-D Scale: A Self-Report Depression Scale for Research in the General Population. Appl. Psychol. Meas..

[73] Hollist C.S., Miller R.B., Falceto O.G., Fernandes C.L. (2007). Marital Satisfaction and Depression: A Replication of the Marital Discord Model in a Latino Sample. Fam. Process.

[74] Sandberg J.G., Yorgason J.B., Miller R.B., Hill E.J. (2012). Family-to-Work Spillover in Singapore: Marital Distress, Physical and Mental Health, and Work Satisfaction. Fam. Relat..

[75] Prestin A., Nabi R. (2020). Media Prescriptions: Exploring the Therapeutic Effects of Entertainment Media on Stress Relief, Illness Symptoms, and Goal Attainment. J. Commun..

[76] Azizan N.H., Mahmud Z. (2018). Determinants of Subjective Well-Being: A Systematic Review. Environ.-Behav. Proc. J..

[77] Mickelson K.D., Claffey S.T., Williams S.L. (2006). The Moderating Role of Gender and Gender Role Attitudes on the Link between Spousal Support and Marital Quality. Sex Roles.

[78] Wilcox W.B., Nock S.L. (2006). What’s Love Got to Do with It? Equality, Equity, Commitment and Women’s Marital Quality. Soc. Forces.

[79] Daspe M.E., Vaillancourt-Morel M.P., Lussier Y., Sabourin S. (2018). Facebook Use, Facebook Jealousy, and Intimate Partner Violence Perpetration. Cyberpsychol. Behav. Soc. Netw..

[80] Elphinston R.A., Noller P. (2011). Time to Face It! Facebook Intrusion and the Implications for Romantic Jealousy and Relationship Satisfaction. Cyberpsychol. Behav. Soc. Netw..

